# The Impact of Diabetic Conditions and AGE/RAGE Signaling on Cardiac Fibroblast Migration

**DOI:** 10.3389/fcell.2020.00112

**Published:** 2020-02-25

**Authors:** Stephanie D. Burr, Mallory B. Harmon, James A. Stewart Jr.

**Affiliations:** Department of BioMolecular Sciences, School of Pharmacy, The University of Mississippi, Oxford, MS, United States

**Keywords:** AGE/RAGE signaling, cardiac fibroblasts, fibroblast migration, diabetes, Rap1a

## Abstract

Diabetic individuals have an increased risk for developing cardiovascular disease due to stiffening of the left ventricle (LV), which is thought to occur, in part, by increased AGE/RAGE signaling inducing fibroblast differentiation. Advanced glycated end-products (AGEs) accumulate within the body over time, and under hyperglycemic conditions, the formation and accumulation of AGEs is accelerated. AGEs exert their effect by binding to their receptor (RAGE) and can induce myofibroblast differentiation, leading to increased cell migration. Previous studies have focused on fibroblast migration during wound healing, in which diabetics have impaired fibroblast migration compared to healthy individuals. However, the impact of diabetic conditions as well as AGE/RAGE signaling has not been extensively studied in cardiac fibroblasts. Therefore, the goal of this study was to determine how the AGE/RAGE signaling pathway impacts cell migration in non-diabetic and diabetic cardiac fibroblasts. Cardiac fibroblasts were isolated from non-diabetic and diabetic mice with and without functional RAGE and used to perform a migration assay. Cardiac fibroblasts were plated on plastic, non-diabetic, or diabetic collagen, and when confluency was reached, a line of migration was generated by scratching the plate and followed by treatment with pharmacological agents that modify AGE/RAGE signaling. Modification of the AGE/RAGE signaling cascade was done with ERK1/2 and PKC-ζ inhibitors as well as treatment with exogenous AGEs. Diabetic fibroblasts displayed an increase in migration compared to non-diabetic fibroblasts whereas inhibiting the AGE/RAGE signaling pathway resulted in a significant increase in migration. The results indicate that the AGE/RAGE signaling cascade causes a decrease in cardiac fibroblast migration and altering the pathway will produce alterations in cardiac fibroblast migration.

## Introduction

Individuals with diabetes mellitus, both type I and type II, are at an increased risk of developing complications as a result of hyperglycemia, such as cardiovascular disease (American Diabetes Association, 2018). A common form of cardiovascular disease associated with diabetics is left ventricle (LV) hypertrophy, which contributes to poor heart function (Fowlkes et al., 2013; Shang et al., 2018). Studies have indicated that myocardial remodeling and fibrosis could be a possible mechanism leading to LV hypertrophy due to cardiac cell-mediated remodeling of the extracellular matrix (ECM) composition (Shang et al., 2018). Fibrosis is a common complication of diabetes, and cardiac tissues from type II diabetics, with no other disease risk factors, have an increased amount of interstitial ECM accumulation (Fowlkes et al., 2013). The increase in fibrosis can be attributed to an increasing number of fibroblasts differentiating into myofibroblasts (Fowlkes et al., 2013).

Cardiac fibroblasts orchestrate the maintenance, synthesis, and degradation of the ECM and can be influenced by both extracellular and intracellular signaling (Lerman et al., 2003; Hutchinson et al., 2013). Among the mechanisms shown to induce myofibroblast transition are matrix metalloproteases (MMPs) and tissue inhibitors of matrix metalloprotease (TIMPS), which collectively can modify the ECM in response to extracellular stimuli (Yabluchanskiy et al., 2013; Caley et al., 2015). These signaling cues can activate neighboring fibroblast cells to differentiate into myofibroblasts to migrate and actively remodel the ECM by increasing matrix protein synthesis and secretion production resulting in fibrosis (Lerman et al., 2003; Darby et al., 2014). In addition, it has been shown that a greater number of myofibroblasts can be detected, via increased levels of α-smooth muscle action (α-SMA), increased cell migration, and increased ECM production, in the heart of diabetic individuals (Shamhart et al., 2014). While we know external circumstances and extracellular signaling can induce myofibroblast differentiation, the mechanism by which these events are triggered is still unclear. One possible mechanism triggering myofibroblast transition could be the AGE/RAGE signaling cascade.

Advanced glycated end-products (AGEs) are formed through a non-enzymatic reaction in which sugar molecules and proteins are combined (Hegab et al., 2012). Accumulation of AGEs occurs naturally in the body and are usually present to a lesser degree in healthy individuals (Simm et al., 2007). AGE accumulation occurs at a higher rate and are more abundant in individuals suffering from elevated glucose levels, such as seen in diabetics (Hegab et al., 2012). The accumulation of AGEs can lead to an increase in crosslinking of matrix proteins, such as collagen, to contribute to the rigidity of the ECM (Ramasamy et al., 2011). In addition, AGEs can elicit an intracellular signaling cascade by activating their receptor (RAGE; receptor for AGEs) (Bierhaus et al., 2005). When activated, the AGE/RAGE cascade stimulates growth factor secretion and increased collagen production along with upregulation of RAGE expression on the cellular membrane (Suchal et al., 2017). A loss of cellular elasticity due in activation of AGE/RAGE signaling affects the physiological function of many tissues and organs. For example, studies have shown that AGE/RAGE signaling can produce endothelial dysfunction by impacting vasodilation in coronary arterioles in type II diabetic mice (Gao et al., 2008). When activated, the AGE/RAGE cascade subsequently stimulates the cardiac fibroblast to myofibroblast transition resulting in increased ECM production or fibrosis leading to the development of cardiovascular disease such as LV stiffness commonly observed in diabetic patients (Suchal et al., 2017). An additional protein thought to impact AGE/RAGE signaling is Rap1a, a small GTPase, which may contribute to activation of cardiac fibroblasts.

The repressor/activator protein 1a (Rap1a) homolog is associated with different organ systems and multiple signaling pathways. Focusing on the cardiovascular system, Rap1a is involved in the development and functionality of the heart (Dong et al., 2012). Rap1a is a member of the Ras superfamily which is composed of small GTPases (Dong et al., 2012). Due to its ability to act as a molecular switch, Rap1a is able to act as a connector to transmit extracellular signaling to intracellular signals (Yan et al., 2008). Rap1a is able to bind and activate an assortment of proteins including cardiovascular effector proteins (Roberts et al., 2013). These effector proteins can regulate mechanisms like cell proliferation, cell adhesion, and cell migration within the cardiovascular tissue (Jeyaraj et al., 2011). Under hyperglycemic conditions, Rap1a has been shown to activate downstream signaling pathways, such as extracellular signal-regulated kinase 1/2 (ERK1/2) (Asif et al., 2002; Yan et al., 2008). Activation of ERK1/2 pathway stimulates ECM fibrosis, which contributes to cardiovascular disorders (Asif et al., 2002). Based off initial studies, Rap1a appears to intersect the AGE/RAGE cascade to promote activation of cardiac fibroblasts, leading to ECM remodeling and consequently fibrosis within the heart.

Migration of cells is an integral part in the structure and function of body systems. Stimuli such as cytokines and growth factors have been shown to induce cardiac fibroblast differentiation into myofibroblasts, which is characterized by increased motility (Li et al., 2004; Velnar et al., 2009; Fowlkes et al., 2013). Currently, there are conflicting research regarding diabetic fibroblast migration. Previous studies have demonstrated an impaired migration in diabetic dermal fibroblasts; however, other studies have reported that hyperglycemic conditions increased rat cardiac fibroblast migration (Chen et al., 1996; Lerman et al., 2003; Straino et al., 2008; Velnar et al., 2009). This project aims to fill in the gaps in knowledge regarding cardiac fibroblast migration in diabetics. More specially, our laboratory aims to examine the effects of the AGE/RAGE signaling cascade on cardiac fibroblast migration in diabetes. Therefore, we hypothesized activation of the RAGE cascade alters cardiac fibroblast migration in type II diabetic mice. Using genotypically different cardiac fibroblasts on collagen matrices from diabetic and non-diabetic mice, we were able to observe changes in cell mobility to determine a mechanistic role for RAGE signaling and Rap1a in diabetes-induced fibroblast migration. The results of this study determined that diabetic fibroblasts exhibited a higher degree of migration compared to non-diabetic fibroblasts. Examination of AGE/RAGE signaling on cardiac fibroblast migration determined that AGE/RAGE signaling lowered the amount of fibroblast migration. Overall, this reduction in migration can be alleviated with prevention or reduction of AGE/RAGE signaling.

## Materials and Methods

### Animal Models

Male *Lepr*^*db*^ (db/db model, referred to as diabetic) type II diabetes mellitus mice (BKS.Cg-*Dock7*^*m*^+/+ *Lepr*^*db*^/J, Jackson Labs) were used for this study. The db/db mouse model has a point mutation in the leptin receptor leading to a nonfunctional leptin receptor. This mutation results in obesity and insulin resistance leading to the development of hyperglycemia by 8 weeks of age and overt diabetes by 12 weeks of age. Heterozygous mice (non-diabetic) were used as lean controls.

Male RAGE knockout mice (RAGE RKO) were used for this study. A Tie2 Cre mouse line was generated by flanking the extracellular domain of the receptor with two *loxP* sites in the same orientation. Additionally, a reversely oriented transcriptional EGFP reporter gene was inserted into intron 1, and a neomycin cassette and a thymidine kinase promoter were inserted into intron 7. EGFP PCR genotyping reactions are performed as a positive control for RAGE knockout mice (Constien et al., 2001; Liliensiek et al., 2004; Brodeur et al., 2014). After exposure to Cre recombinase (*Cre*), the *loxP* flanked sequences were deleted, resulting in the global loss of RAGE mRNA expression and loss of RAGE signaling. RAGE knockout mice were crossbred with heterozygous (non-diabetic) mice to generate RAGE knockout diabetic (diabetic RKO) and non-diabetic (non-diabetic RKO) mice (Constien et al., 2001; Liliensiek et al., 2004). Breeder mice were a generous gift from Dr. Pamela Lucchesi and Dr. Angelika Bierhaus.

Male Rap1a knockout mice (Rap1a KO) and wild-type (Rap1a WT) were used for this study. This mouse model was generated by inserting a neomycin resistant gene downstream of exon 4 of RAP1A in the opposite (3′–5′) orientation. A targeting vector (a 0.95 kb *PvuII-NdeI* fragment) was used to insert the resistance gene in order to disrupt Rap1a mRNA expression (Li et al., 2007). Breeder mice were a generous gift from Dr. Maqsood Chotani and Dr. Lawerence Quilliam.

### Animal Care

All experiments were performed using adult male mice at 16 weeks of age. The mice were housed under standard environmental conditions and maintained on commercial mouse chow and tap water *ad libitum*. All studies conformed with the principles of the National Institutes of Health “Guide for the Care and Use of Laboratory Animals,” (NIH publication No. 85-12, revised 1996), and the protocol was approved by the University of Mississippi Institutional Animal Care and Use Committee (protocol #17-024). Anesthesia for euthanasia at the experimental endpoint was caused by carbon dioxide inhalation followed by cervical dislocation, which served as a second mechanism for euthanasia. At this time, the chest was opened, and the heart was quickly excised for further cellular, histological, and biochemical experiments.

### Fibroblast Isolation and Cell Culture

Mice were weighed and non-fasting blood glucose levels were measured via tail cut (Table 1: glucometer; OneTouch Ultra^®^, LifeScan, Inc., Johnson & Johnson). Hearts were then removed from the chest cavity, the atria dissected away, and ventricles weighed (Table 1). Three hearts of each genotype were used for a single fibroblast isolation. In a sterile, cell culture hood, the hearts were cut into ∼5 mm small pieces and placed a 10 mL of collagenase-trypsin enzymatic solution (0.1% Trypsin, Gibco; 100 U/mL collagenase II, Worthington Biochemical). Fibroblasts were continually mixed in the tissue-enzymatic mixture using water-jacketed spinner flasks maintained at 37°C until hearts were broken down into a single cell suspension. The single cell suspension was centrifuged (250 x *g* at room temperature for 10 min) and resuspended in high glucose Dulbecco’s Modified Eagles Medium (DMEM) [high glucose media; DMEM containing 4.5 g/L glucose, sodium pyruvate, L-glutamine, and supplemented with 14.2 mM NaHCO_3_, 14.9 mM HEPES, 30% heat-inactivated fetal bovine serum (FBS), 1% L-glutamine, and 0.02% Primocin (Thermo Fisher)] for 24 h in an incubator buffered with 5% CO_2_ kept at 37°C. After 24 h, the cardiac fibroblasts were washed with their appropriate media three times and then incubated at 37°C in their appropriate media [non-diabetic and Rap1a fibroblasts: low glucose (1 g glucose/L) and diabetic fibroblasts (these are fibroblasts removed from diabetic mouse hearts): high glucose (4.5 g glucose/L)]. All experiments were performed using cells at P1, in order to ensure the cell *in vivo* phenotype was maintained. Cells were passaged just prior to reaching 95% confluency using a 0.25% trypsin and 0.1% ethylenediaminetetraacetic acid (trypsin/EDTA) solution (Life Technology). Both cell culture and migration plates were kept in a CO_2_ incubator at 37°C.

**TABLE 1:** Physiological measurements of mice.

	**Body weight (g)**	**Blood glucose (mg/dL)**	**Heart weight (g)**
Non-diabetic (*n* = 47)	29.05 ± 0.37	204.7 ± 7.30	0.1173 ± 0.005
Diabetic (*n* = 28)	51.09 ± 1.26****	525.0 ± 22.67****	0.1106 ± 0.002
Non-diabetic RKO (*n* = 41)	32.32 ± 0.43***	213.3 ± 4.58	0.1184 ± 0.002
Diabetic RKO (*n* = 12)	56.61 ± 0.70****	412.7 ± 29.44****	0.1216 ± 0.002
Rap1a WT (*n* = 34)	27.72 ± 0.35	217.2 ± 6.32	0.1106 ± 0.002
Rap1a KO (*n* = 16)	27.61 ± 0.74	172.1 ± 6.73	0.1085 ± 0.005

Data represents average values from mice used for cardiac fibroblast isolation with an average of three hearts per isolation. A one-way ANOVA was conducted followed by a Tukey’s *post hoc* test to determine significant difference between non-diabetic mice and the other genotype groups (*** *p* < 0.001, **** *p* < 0.0001).

### Collagen Extraction

Tails from non-diabetic and diabetic mice were collected and stored at −20°C. After roughly 20 tails had been collected, the four major tail tendons were removed, minced, washed in dH_2_O, and placed in 150 mL acetic acid (1:1000 dilution in dH_2_O). The tendons were continually mixed for 3 days at 4°C (Rajan et al., 2007). All steps, hereinafter, were conducted in sterile conditions in a cell culture hood. After 3 days, the tendon-acetic acid solution was centrifuged at 3000 x *g* for 30 min at 4°C. The supernatant, containing the collagen, was removed to a new container and centrifuged again under the same conditions. The collagen solution was stored at 4°C, and concentration was estimated using Sircol^TM^ Soluble Collagen Assay Kit (BioColor Ltd.) as per manufacturer’s directions.

### Western Blot Analysis

Protein was isolated from cells cultured in 60 mm dish using modified Hunter’s buffer [MHB; 1% Triton X-100, 75 mM NaCl, 5 mM tris pH 7.4, 0.5 mM orthovanadate, 0.5 mM ECTA, 0.5 mM EGTA, 0.25% NP-40, and freshly added Halt Protease Inhibitor Cocktail (100×; Thermo Fisher)]. Plates were incubated on ice with MHB for 10 min then a cell scrapper was used to dislodge cells from plate. Cell lysate was removed and placed into 1.5 mL tube followed by probe-sonication. Samples were centrifuged for 15 min at 32,000 x *g* at 4°C and supernatant was removed and stored at −80°C. Protein concentration was determined using a bicinchoninic acid assay (BCA; Pierce Biotechnology) according to manufacturer’s instructions. Twelve micrograms of protein was used per sample for western blot analysis. Antibodies used were as follow: monoclonal α-smooth muscle actin [α-SMA (1:400); Sigma Aldrich 2547], RAGE [(1:400) Santa Cruz sc-365154], and β-tubulin [(1:400) Santa Cruz sc-398937] were used as a loading control. Western blots were visualized using an iBRIGHT imaging system.

### Migration Assay

Migration assay plates consisted of a 48-well culture plate with a line and hash marks drawn on the bottom of each well for image orientation. Before plating cells, 50 μL non-diabetic and diabetic collagen were added to wells and incubated for 1 h at 37°C (Lerman et al., 2003). Excess collagen was removed with 1× sterile PBS wash. Next, fibroblasts at P1 were plated and grown to 95% confluency. The experimental design for assessing migration used two replicates per treatment. Once reaching confluency, the media was suctioned off and each well was scratched with the tip of a 200 μL pipet tip along the previously marked line through the center of each well on the bottom of the plate (Xuan et al., 2014). After scratching, the plates were rinsed with the appropriate media containing 1.5% FBS. Initial experiments conducted indicated a concentration of 1.5% FBS promoted cell migration and at the same time prevent cell division. 500 μL of the appropriate 1.5% FBS media was added to each well. At this time, AGE-BSA (glycated albumin 0.5 mg/mL), U0126 (5 μm; inhibitor of ERK), and PKC-ζ pseudosubstrate (1 μg/mL; ps-PKC-ζ) were added. U0126 and PKC-ζ pseudosubstrate were selected as inhibitors for the AGE/RAGE cascade due to previous studies indicating these proteins are involved in the AGE/RAGE cascade (Wu et al., 2003; Adamopoulos et al., 2016; Evankovich et al., 2017). The cells were allowed to incubate for 24°h after scratching. Images of cells were taken at 0 and 24°h post scratching.

### Immunohistology

Morphological evaluation of collagen content was performed on hearts from age-matched Rap1a WT and KO mice as previously described (Stewart et al., 2003). Briefly, hearts were fixed in histology grade 4% paraformaldehyde and embedded in paraffin blocks. Blocks were sectioned at 5-μm thickness from the equator of the heart and stained with Picric Acid Sirius Red F3BA. Estimates of the fractions of thick and thin collagen fibrils were obtained by using polarized light. Due to the birefringent quality of the stain, collagen refracted a distinct color based upon the size of the collagen fibrils: red and yellow (thick filaments) and green (thin filaments). Quantitative analysis is accomplished by light microscopy with a video-based image-analyzer system. Color thresholds were set for biphasic analysis to capture and generate a percent collagen content per 40× field within the specified RGB wavelength ranges separate from the background: Phase 1 represents collagen capture Red (0–40) Green (0–80) Blue (0–255) and Phase 2 represents background capture: Red (20–255) Green (40–255) Blue (35–255). Results are presented as the mean ± SEM values computed from the average of *n* = 25–35 individual measurements obtained from each heart. Cardiac vasculature, epicardium, and endocardium were avoided due to high levels of collagen content and do not accurately reflect myocardial interstitial collagen.

Non-diabetic and diabetic collagen were fixed with histology grade 4% paraformaldehyde for 10 min at room temperature. Collagen was then incubated with blocking solution (3% donkey serum, 2% BSA, and 0.01% Triton X-100 in 1× PBS) overnight at 4°C. After 24 h, primary antibody was added and incubated overnight at 4°C. AGE antibody (1:50; Abcam) and carboxymethyl lysine (CML) antibody (1:50; Abcam) were added to collagen. Slides were mounted using Vectashield hard set mounting media.

### Cell Migration Imaging and Analysis

At the 0 h time point, pictures were taken to document the position of cells prior to migration using a Zeiss Primovert microscope with camera (Zen Blue 2.3 edition, Zeiss). Hashmarks along the scratch line were used to align the plate to determine areas of interest for imaging as well as were used to prevent imaging of overlapping regions. At the 24 h time point, the plate was removed from the incubator and fixed with 4% paraformaldehyde fixative for 10 min at room temperature. After 10 min, the fixative was removed, and the cells were washed with 1× PBS. Next, cells were incubated in 1× PBS with 0.1% Trition-100 for 30 min at room temperature with continuous rocking motion to permeabilize the fixed cells. Cells were stained with 1% Brilliant Blue Coomassie stain (3% Coomassie Brilliant Blue, 10% acetic acid, 45% methanol, and 45% dH_2_O) for 10 min at room temperature with continual rocking. Finally, 24 h post-scratched pictures of the cells were taken using the 0 h time point images as templates for the correct position of the areas of interest.

Adobe Photoshop Elements 2018 was used to draw lines on 0 h images to denote the scratched area. The scratched areas were determined by denoting the linear area that lacked fibroblasts. Areas that lacked fibroblasts, but where not in line with the orientation of the scratch, were not included in the calculation as these areas may contain cells which detached from the culture dish for reasons other than the physical scratch. The 0 h image was superimposed on the 24 h image in order to indicate the scratch area (i.e. line of migration) on the 24 h image. Next, the number of cells that had migrated across the lines drawn were counted and recorded for each well. The area of the scratch was calculated using Image J, and the number of migrated cells was normalized to calculated area of the scratch by dividing the number of migrated cells by the area. This process was completed twice for two separate images which together showed approximately the total cell migration of one well.

### Statistical Analysis

Differences in non-diabetic and diabetic, non-diabetic RKO and diabetic RKO, and Rap1a WT and Rap1a KO migration on different collagens were determined by performing a Student’s *t*-test. Differences associated with inhibition of RAGE signaling were assessed by one-way analysis of variance (ANOVA). After one-way ANOVA analysis, a Dunnett’s *post hoc* test was conducted in order to determine differences between control and treatment groups. Comparisons between genotypes and AGE-treated fibroblasts were determined by performing two-way ANOVA. A Sidak’s *post hoc* test was conducted in order to assess differences between different genotypes on the same type of collagen as well as differences between the control and AGE-treated fibroblasts of the same genotype. GraphPad Prism 8 was used for all statistical analysis. Significance was defined as *p* < 0.05. Error bars represent ±standard error of the mean (SEM).

## Results

### The Presence of RAGE Signaling Negatively Impacts Cardiac Fibroblast Migration

**FIGURE 1:**
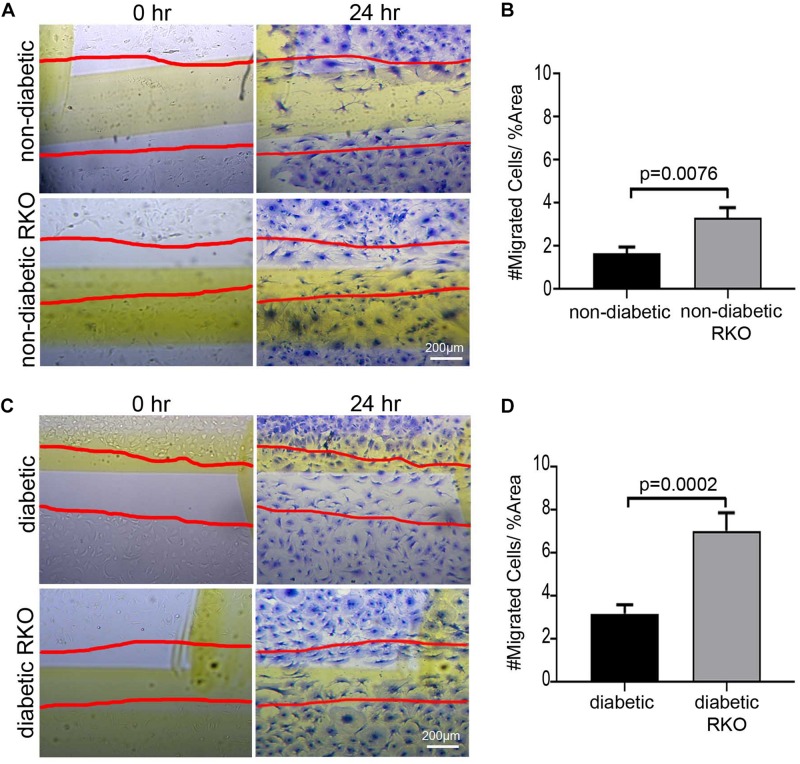
The presence of RAGE signaling negatively impacts cardiac fibroblast migration. Cardiac fibroblasts were isolated from **(A,B)** non-diabetic with and without RAGE mice as well as **(C,D)** diabetic with and without RAGE mice. Fibroblasts were plated onto plastic cell culture dishes, scratched (0 h: **A,C**), and assessed for cell migration after 24 h **(B,D)**. Cells were stained with Coomassie for visualization with the red lines depicting scratched area (40× and scale bar = 200 μm). Number of migrated cells were normalized to percent scratched area and plotted as mean ± SEM (*n* = 7–13). Student’s *t*-test used to determine significance, which is depicted on graph.

Isolated cardiac fibroblasts were used to assess the impact of RAGE signaling on cell migration (Figure 1). Prevention of RAGE signaling by genetically altering RAGE to a non-functioning receptor (RAGE knockout, RKO) resulted in increased non-diabetic RKO fibroblast (3.31 #migrated cells/% area) migration over that of non-diabetic fibroblasts (1.66 #migrated cells/% area) (Figures 1A,B; Student’s *t*-test *p* = 0.0076). Diabetic RKO fibroblasts (7.04 #migrated cells/% area) migrated significantly more than diabetic fibroblasts (3.19 #migrated cells/% area) (Figures 1C,D; Student’s *t*-test *p* = 0.0002). Diabetic fibroblasts, regardless of the presence or absence of RAGE, migrated significantly more than non-diabetic fibroblasts with percent differences ranging from 63% between non-diabetic and diabetic to 73% for RKO non-diabetic versus RKO diabetic fibroblasts. Further studies were then performed to determine the role of Rap1a in AGE/RAGE signaling and fibroblast migration.

### Knockout of Rap1a Results in Decreased Collagen Expression in the Heart as Well as Reduced Protein Expression of Specific AGE/RAGE Markers

**FIGURE 2:**
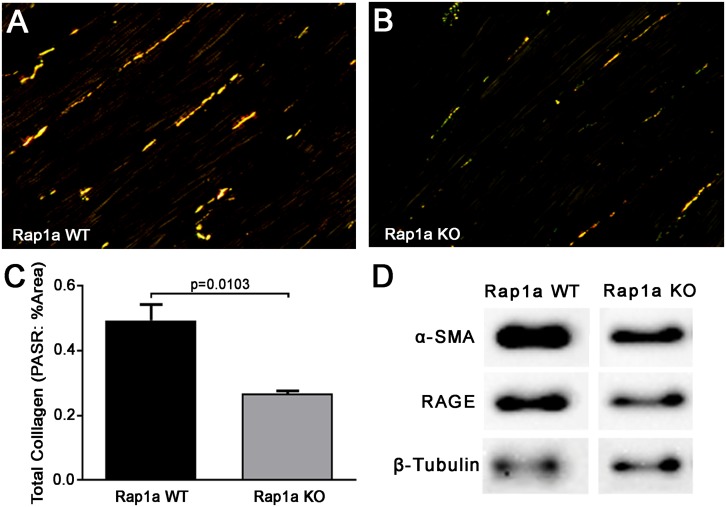
Knockout of Rap1a resulted in decreased collgen expression in hearts (*in vivo*) and reduced protein expression of specific AGE/RAGE signaling markers in *cardiac fibroblasts* (*in vitro*). Hearts from Rap1a wild-type (WT; **A**) and knockout (KO; **B**) mice were sectioned and stained with picric acid sirius red (PASR) which fluorescences with exposure to polaized light. Total collagen was determined by normalizing the amount of PASR fluorescence to the percent area and graphed as mean ± SEM **(C)**. Student’s *t*-test was preformed to assess significance with a sample size of three to four hearts. Protein expression for specific AGE/RAGE signaling cascade indicators was assessed in cardiac fibroblasts isolated from Rap1a WT and KO hearts **(D)**. Alpha smooth muscle actin (α-SMA) was used to indicate the presence of differentiated fibroblasts (myofibroblasts) and RAGE indicated expression of the receptor for AGE (RAGE). β-Tubulin was used as a loading control. Western blot images shown are not displayed as a continuous blot due to running on different membranes.

Previous studies by our laboratory have revealed that silencing Rap1a in diabetic cells altered RAGE protein expression (Zhao et al., 2014). By manipulating Rap1a, we could amplify or reduce the RAGE signaling pathway to modify signaling outcomes, accordingly. We further examined total collagen expression, an outcome of the AGE/RAGE cascade, in hearts from Rap1a wild-type (Rap1a WT) and Rap1a knockout (Rap1a KO) mice (Figures 2A–C). Rap1a KO hearts had significantly less total collagen compared to Rap1a WT hearts (Figure 2C, Student’s *t*-test *p* = 0.0103). Further examination of AGE/RAGE signaling outcomes in cardiac fibroblasts isolated from Rap1a WT and KO hearts indicated a decrease in α-SMA and RAGE protein expression, indicative of reduced AGE/RAGE signaling (Figure 2D). Due to Rap1a impact on AGE/RAGE signaling outcomes *in vivo* and *in vitro*, we examined the impact of Rap1a on cardiac fibroblast migration.

**FIGURE 3:**
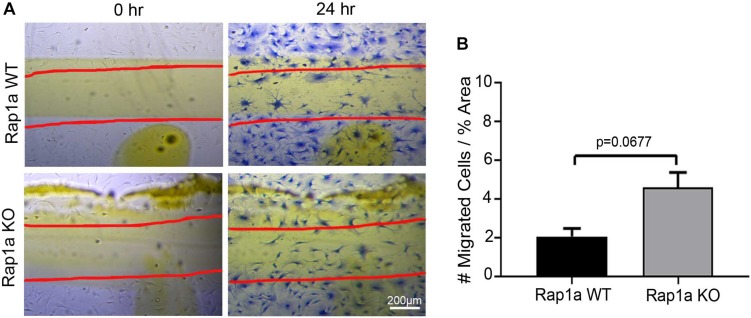
Knockout of Rap1a led to an increase in cardiac fibroblast migraiton. Cardiac fibroblasts were isolated from Rap1a WT and KO hearts, cultured on plastic cell culture dish, scratched (0 h) and assessed for migration after 24 h **(A)**. Scratched lines were depicted with red lines and cells were stained with Coomassie blue for visualization (40× and scale bar = 200 μm). Number of migrated cells were normalized to percent scratched area and graphed as mean ± SEM (**B**; *n* = 6–10). Statistical anayslsis consisted of Student’s *t*-test.

To further investigate this idea, cardiac fibroblasts from Rap1a KO and Rap1a WT mice were used to determine if changes in Rap1a expression effected changes in the RAGE signaling cascade to impact fibroblast migration. Rap1a KO fibroblasts (4.62 #migrated cells/% area) tended to have more migration when compared to Rap1a WT fibroblasts; even though, results were not significant (2.09 #migrated cells/% area) (Figures 3A,B; Student’s *t*-test *p* = 0.0677). Rap1a WT fibroblasts (2.09 #migrated cells/% area) had similar migration rates compared to non-diabetic fibroblasts (1.66 #migrated cells/% area). Whereas Rap1a KO fibroblast migration was similar to that of non-diabetic RKO fibroblasts. The percent difference between Rap1a KO and WT fibroblasts was 76% which is comparable to RKO fibroblasts. These results indicate that with reduced RAGE signaling or elimination of RAGE, cardiac fibroblast migration was shown to be increased.

### The Elevated Levels of AGEs in Diabetic Collagen Did Not Cause a Significant Impact on Fibroblast Migration

**FIGURE 4:**
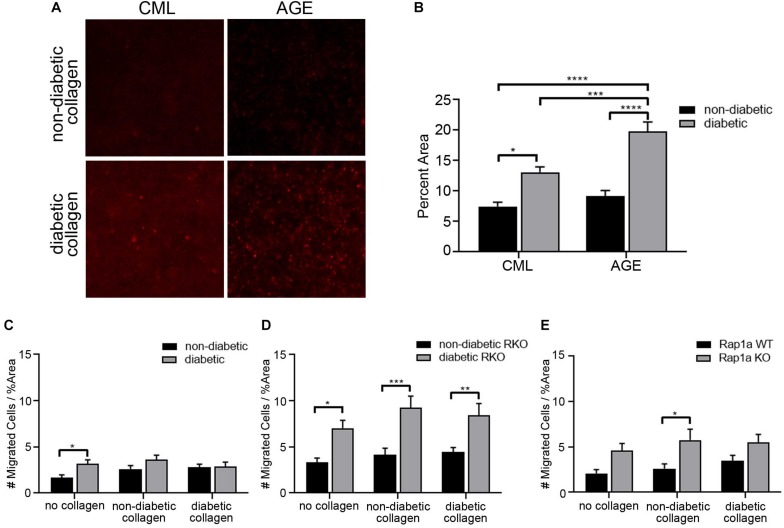
The elevated levels of AGEs in diabetic collagen did not significantly impact fibroblast migration. **(A)** Immunofluorescence (100×; scale bar = 50 μm) images for carboxymethyl lysine (CML) and advanced glycated end-products (AGEs) in non-diabetic collagen and diabetic collagen. **(B)** Graph depicting semi-quantification of CML and AGEs present in non-diabetic and diabetic collagen. Cardiac fibroblasts isolated from **(C)** non-diabetic and diabetic, **(D)** non-diabetic and diabetic RAGE knockout, and **(E)** Rap1a WT and KO mice were plated on either no collagen (results shown in Figure 1) non-diabetic collagen, or diabetic collagen. The number of migrated cells were normalized to percent scratch area. Data are mean ± SEM with a two-way ANOVA and a Sidak’s *post hoc* test, *n* = 10–13. A red line was set at *y* = 1 Migrated Cells/%Area and was used for comparison across cardiac fibroblast genotypes (* *p* < 0.05, ** *p* < 0.01, *** *p* < 0.001, **** *p* < 0.0001).

In an *in vivo* setting, cardiac fibroblasts undergoing migration will continuously interact with the ECM, and the composition of the matrix will conversely impact cell behavior. In order to replicate this environment, specially focusing on RAGE signaling, collagen from diabetic and non-diabetic mice was used as a scaffold for migration assays. Extracted tail collagen from diabetic mice had higher accumulation of CMLs and AGEs compared to non-diabetic collagen (Figure 4A). Based off these findings, collagen could be used to assess changes in fibroblast migration. Cells were plated on plastic, which had no AGEs, on non-diabetic collagen, which had a very low presence of AGEs, and diabetic collagen, which had high levels of AGEs, to determine the impact of AGE accumulation and presence on fibroblast migration.

The difference in the amount of AGEs in collagen did not significantly impact the number of migrated fibroblasts. Non-diabetic fibroblast migration slightly increased as the levels of AGEs rose from no AGEs on plastic, to low AGE levels on non-diabetic collagen, and to high concentrations of AGEs on diabetic collagen. In contrast, diabetic fibroblast migration did not change between plastic, non-diabetic collagen, and diabetic collagen (Figure 4C; two-way ANOVA, genotype *p* = 0.0094). In contrast, non-diabetic RKO and diabetic RKO fibroblasts displayed similar amounts of migration with increasing accumulation of AGEs from plastic to diabetic collagen (Figure 4D; two-way ANOVA, genotype *p* < 0.0001). Rap1a WT and Rap1a KO fibroblasts displayed similar migration when plated on plastic, non-diabetic, and diabetic collagen (Figure 4E; two-way ANOVA, genotype *p* = 0.0005).

### Cardiac Fibroblasts Display Increased Migration With Attenuated RAGE Signaling on Diabetic Collagen

**FIGURE 5:**
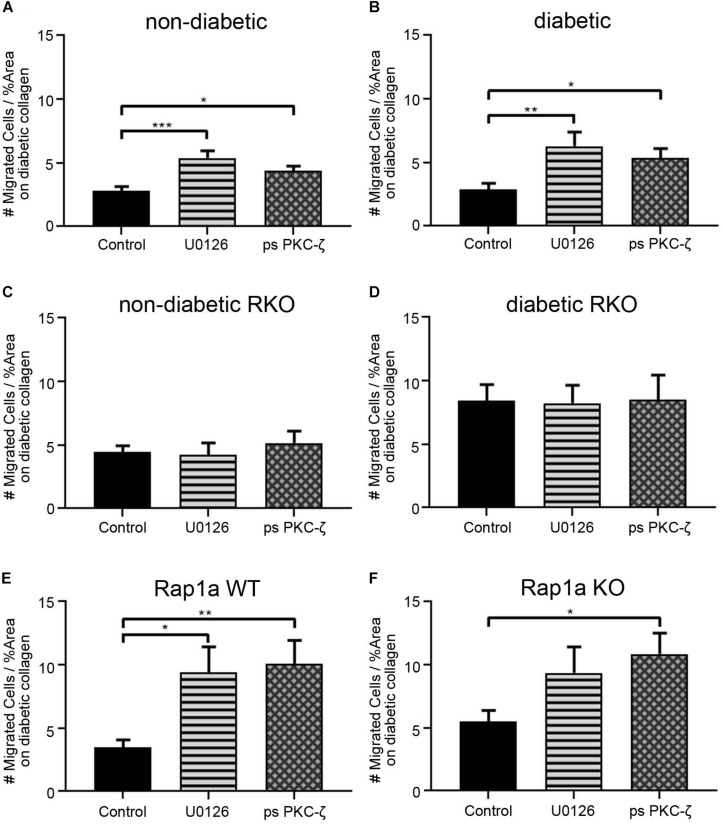
Cardiac fibroblasts display increased migration with decreased RAGE signaling on diabetic collagen. **(A)** Non-diabetic, **(B)** diabetic, **(C)** non-diabetic RKO, **(D)** diabetic RKO, **(E)** Rap1a WT, and **(F)** Rap1a KO cardiac fibroblasts plated on diabetic collagen were treated with U0126 (ERK inhibitor; 5 μM) and ps PKC-ζ (pseudosubstrate PKC-ζ inhibitor; 1 μg/mL). Data represent mean ± SEM with a *n* = 10–13 for control and *n* = 3–9 for treatments. A one-way ANOVA and a Dunnett’s *post hoc* test determined significance (* *p* < 0.05, ** *p* < 0.01, *** *p* < 0.001).

In order to further demonstrate the impact of AGE/RAGE signaling on migration, U0126 (ERK inhibitor, 5 μM) and pseudosubstrate PKC-ζ (ps PKC-ζ, PKC-ζ inhibitor, 1 μg/mL) were used to dampen RAGE signaling in cardiac fibroblasts. Fibroblast migration on diabetic collagen was used, as diabetic collagen contained the higher prevalence of AGEs and would allow for the best insight into the impact of AGE/RAGE signaling on migration (Figure 5). Non-diabetic fibroblasts migrated significantly more when treated with U0126 and ps PKC-ζ (Figure 5A; one-way ANOVA *p* = 0.0004) when compared to untreated non-diabetic cells. Similar increased levels of migration occurred with treated diabetic fibroblasts (Figure 5B; one-way ANOVA *p* = 0.0058). In contrast, fibroblasts lacking functional RAGE (RKO fibroblasts) did not exhibit altered cell migration when treated with either U0126 or ps PKC-ζ (Figures 5C,D; one-way ANOVA, non-diabetic RKO *p* = 0.8595, diabetic RKO *p* = 0.9927).

Rap1a WT cardiac fibroblasts displayed a significant increase in migration with U0126 and ps PKC-ζ treatment (Figure 5E; one-way ANOVA *p* = 0.0033). Although Rap1a KO fibroblasts had increased migration with U0126 and ps PKC-ζ treatment, only ps PKC-ζ was significant (Figure 5F; one-way ANOVA *p* = 0.0303).

### Increased Exogenous AGEs in Diabetic Collagen Led to Increased Fibroblast Migration

**FIGURE 6:**
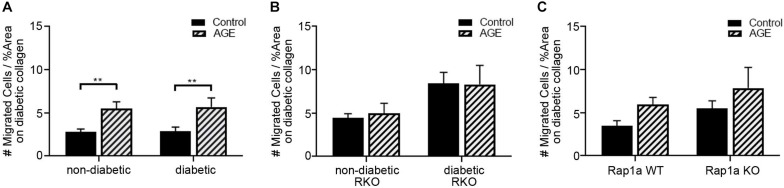
Increased exogenous AGEs in diabetic collagen led to increased fibroblast migration. Cardiac fibroblasts from **(A)** non-diabetic and diabetic, **(B)** RAGE knockout, and **(C)** Rap1a mice were plated on diabetic collagen. Fibroblasts were either untreated (control) or treated with AGEs (0.5 mg/mL). Cell migration was normalized to percent scratch area with mean ± SEM being depicted on graph. Two-way ANOVA with Sidak’s *post hoc* test determined significance (** *p* < 0.01, *n* = 5–10).

While AGEs in the ECM can induce AGE/RAGE signaling, they can also increase stiffness of ECM due to formation of crosslinks between collagen fibers. In order to determine the impact of AGEs on fibroblast migration, additional exogenous AGEs (glycated albumin; 0.5 mg/mL) were added to fibroblasts plated on diabetic collagen (Figure 6). Non-diabetic and diabetic fibroblasts migrated significantly more with treatment of AGEs, compared to untreated control (Figure 6A; two-way ANOVA treatment *p* < 0.0001, Sidak’s *post hoc* test *p* = 0.0061 non-diabetic and *p* = 0.0085 diabetic). Exogenous AGE treatment of RKO fibroblasts did not induce a change in migration (Figure 6B: two-way ANOVA treatment *p* = 0.8549).

Rap1a WT and KO fibroblast migrations were more significantly impacted with AGE treatment (Figure 6C; two-way ANOVA, treatment *p* = 0.0490). These results indicated that AGEs present in the diabetic matrix when combined with the treatment of exogenous AGEs resulted in a shift in fibroblast phenotype to that of a diabetic cell. Diabetic cardiac fibroblasts exhibit significantly more migration compared to their non-diabetic counterparts.

## Discussion

The aim of this study was to assess the role of AGE/RAGE signaling on cardiac fibroblast migration in diabetic conditions. While fibroblast migration, specifically epithelial fibroblast migration, has been widely studied in conjunction with impaired diabetic skin wound healing (Stewart et al., 2010; Darby et al., 2014; Tracy et al., 2014). The mechanism of fibroblast migration in other organ systems under diabetic conditions is still relatively uncertain. The results presented in this study demonstrated diabetic cardiac fibroblasts migrated significantly more than non-diabetic cardiac fibroblasts. By altering either (1) RAGE expression using RKO fibroblasts, (2) suppressing key components of the AGE/RAGE cascade using pharmacological inhibitors, or (3) downregulating RAGE signaling in Rap1a KO cells, we observed an increase in fibroblast migration over that of non-diabetic and diabetic fibroblasts with functional RAGE. These findings contrast those observed in skin fibroblast studies, in that, diabetic cardiac fibroblasts have a higher migration phenotype than non-diabetic cardiac fibroblasts, and by impeding the RAGE signaling cascade, fibroblast migration greatly improved cell migration (Almeida et al., 2016). Thus, providing evidence that decreasing RAGE signaling could potentially improve wound healing in the heart; however, further experiments will need to be performed to determine if this response exists *in vivo*. The AGE/RAGE signaling pathway has been shown to increase ECM deposition; therefore, it could be proposed that the AGE/RAGE signaling cascade may impact cell migration via ECM remodeling (Asif et al., 2002; Yan et al., 2008).

The cardiac ECM is composed of a variety of biological molecules capable of altering cell-matrix interactions resulting in changes in migration phenotype (Charras and Sahai, 2014). It has been demonstrated that diabetics have increased AGE accumulation in their cells as well as in their extracellular compartments (Hartog et al., 2007). AGEs will form crosslinks with one another, which can contribute to a stiffer collagen matrix (Verzijl et al., 2002). Therefore, this study also aimed to determine if collagen isolated from non-diabetic mice with low amounts of AGEs and collagen from diabetic mice with high amounts of AGEs could alter cardiac fibroblast migration. Our results showed that non-diabetic cardiac fibroblasts migrated slightly more as the prevalence of AGEs climbed. These data suggested the composition of the ECM along with the escalating levels of ECM AGEs were a contributing factor impacting cardiac fibroblast migration.

Elevated diabetic cardiac fibroblast migration, as presented in this work, in comparison to other studies, could be attributed to the heterogeneity of fibroblasts. Cells isolated from differing tissue sources will retain their *in vivo* characteristics while in culture, and would thus yield different phenotypic responses to similar stimuli (Sorrell and Caplan, 2004; Driskell and Watt, 2015; Sriram et al., 2015). Hyperglycemic conditions could possibly contribute to changes noted in previous studies in which diabetic cardiac fibroblast migration was examined. Findings have shown that culturing cells in high glucose media can result in changes in cardiac fibroblast migration (Kanazawa et al., 2010; Xuan et al., 2014). Hyperglycemic conditions have also been demonstrated to cause AGE accumulation. While AGEs accumulate naturally within the body over time, the formation and accumulation of AGEs is accelerated under diabetic conditions (Hegab et al., 2012). It has also been noted that AGE serum levels in diabetics are almost double of that of healthy individuals (Hegab et al., 2012). Therefore, it is a strong possibility that fibroblasts residing in distinct tissues could respond differently under diabetic conditions.

**FIGURE 7:**
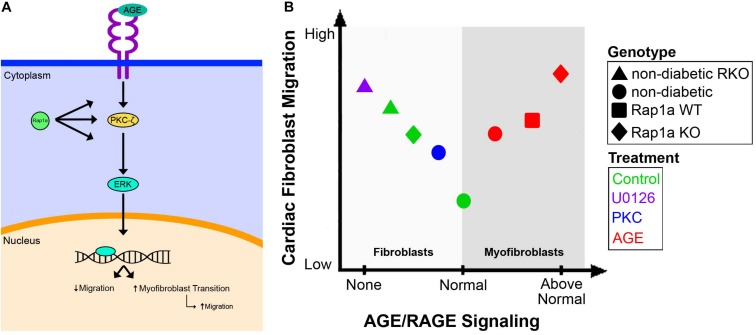
Overview of the AGE/RAGE signaling cascade and its impact on cardiac fibroblast migration. A diagram depicted the proposed signaling network of AGE/RAGE that impacts cardiac fibroblast migration **(A)**. The impact of varying levels of AGE/RAGE signaling on cardiac fibroblast migration and possible differentiation **(B)**.

By studying the effects of the AGE/RAGE signaling pathway on cardiac fibroblast migration, we were able to determine that activation of the signaling cascade altered cardiac fibroblast migration. Fibroblast migration was elevated in diabetic hearts compared to that of cells from non-diabetic hearts. In addition, it was found that cardiac fibroblasts lacking functional RAGE (RKO; RAGE knockout mice) had higher migration levels in both non-diabetic RKO and diabetic RKO fibroblasts when compared to cardiac fibroblasts with intact RAGE. Our results showed when RAGE was eliminated, migration levels were significantly elevated allowing for a more motile cell phenotype, as seen in Figure 7. Additionally, pharmacological inhibition of key signaling proteins within the RAGE cascade, such as ERK1/2 and PKC-ζ, resulted in increased migration (Figure 7A). Migration was not altered by ERK1/2 or PKC-ζ inhibition in RKO fibroblasts, which stands to reason, as the RAGE receptor has been rendered ineffective by genetic ablation. Therefore, downregulating the RAGE cascade allowed for increased cell migration. Due to the extensive impact of ERK1/2 activation in cell signaling, it could be argued that increased migration though inhibition of ERK1/2 was a result of impacting additional pathways other than AGE/RAGE. To counter this argument, an independent study found that inhibition of ERK1/2 activation downstream of MAP kinase signaling resulted in decreased migration (Mitchell et al., 2006). Therefore, if ERK1/2 was influencing migration via a pathway separate from AGE/RAGE, a decrease in migration would be expected. Our results showed increased migration with inhibition of active ERK1/2 to suggest inhibition of AGE/RAGE signaling in cardiac fibroblasts can increase and improve cell migration within the heart.

At basal levels non-diabetic cardiac fibroblasts displayed a lowered migration ability; however, when AGE/RAGE signaling was increased, as in diabetic fibroblasts, there was a concomitant increase in cardiac fibroblast migration. Our study found that treatment with exogenous AGEs also caused an increase in cardiac fibroblast migration much like that observed in diabetic cells, but the level of migration never superseded the migration noted in RKO cardiac fibroblasts. The increase in migration with AGE treatment is most likely due to the cardiac fibroblasts differentiating into myofibroblasts, which can indirectly impact cell migration (Suchal et al., 2017; Figure 7B). Prior research by our lab and others have shown that increases in α-SMA expression, a marker for myofibroblasts, in lung fibroblasts was linked to increased migration (Kawamoto et al., 1997). In addition, in cardiac fibroblasts a decrease in α-SMA expression was correlated with decreased fibroblast migration (Shi et al., 2011). These results support the idea that RAGE signaling above “normal” levels results in increased migration due to these cells transitioning into myofibroblasts, and therefore characterized by elevated migration (Figure 7); however, further studies will need to be performed to determine if AGE can directly induce fibroblast to myofibroblast conversion. Another factor our lab has found to impact RAGE signaling and in turn cardiac fibroblast migration is the small GTPase, Rap1a.

Rap1a acts as a linker molecule connecting extracellular stimuli to intracellular effects. Based off previous findings, we proposed that Rap1a intersects the AGE/RAGE pathway and modifies the signal transduction strength (Yan et al., 2008; Zhao et al., 2014). Our results also demonstrated decreased collagen levels in Rap1a KO hearts as well as reduced protein expression of specific RAGE cascade markers in cardiac fibroblasts. In terms of the role Rap1a plays in fibroblast migration, previously conducted studies showed conflicting results. In colonic epithelial cells decreased Rap1a expression resulted in decreased migration, whereas in macrophages a loss of Rap1a produced increased migration (Li et al., 2007; Severson et al., 2009). Another study by Yan et al. (2008) found that depletion of Rap1a via silencing RNA reduced migration in human microvascular endothelial cells during angiogenesis (Yan et al., 2008). These conflicting studies suggest that the impact Rap1a on cell migration is tissue and/or cell specific. Our study found cardiac fibroblasts without Rap1a (Rap1a KO) had more migration than fibroblasts with Rap1a (Rap1a WT). Comparing this to fibroblasts with functional RAGE and those without RAGE, Rap1a KO cardiac fibroblasts exhibited an intermediate migratory ability. The intermediate migration exhibited in Rap1a KO suggested that Rap1a may contribute to further exacerbation of downstream outcomes of AGE/RAGE signaling to impact cardiac fibroblast migration. Further studies will need to be performed to understand the role of Rap1a in fibroblast function.

The findings of this study suggested that cardiac fibroblast migration is differentially regulated by a number of factors. We found that diabetic fibroblasts have a higher migration level than non-diabetic cells. In addition, the RAGE signaling cascade leads to reduced migration compared to higher migration observed in RKO fibroblasts. Reduction of AGE/RAGE signaling with U0126 and ps-PKC resulted in increased cardiac fibroblast migration. With the addition of AGEs, cell migration was elevated due, most likely, to myofibroblast differentiation brought on by higher concentration of AGEs and increased AGE/RAGE signaling. Decreased RAGE signaling via knocking out Rap1a GTPase resulted in an increase in cardiac migration, but not to the level noted in RKO fibroblasts. Therefore, changes in fibroblast migration and factors that influence this behavior can have the potential to alter organ performance. For example, increased LV collagen deposition by myofibroblasts can impact ventricular remodeling, heart function, and contribute to cardiovascular disease (Zhang et al., 2007; Dludla et al., 2017). As scratch assays may mimic an injury to confound experimental conditions, future studies will need to be performed using a more specific migration, such as Boyden chamber assays, to confirm results. Examination of changes in migration protein markers in the future will provide further evidence to the role of RAGE signaling has on fibroblast migration. In addition to these studies, experiments that assess the impact of diabetic conditions and the link with RAGE signaling can be done to determine the influence of diabetes on cardiac fibroblast migration. While there is still more to investigate regarding AGE/RAGE signaling on cardiac fibroblast migration, our study provides convincing results demonstrating that diabetes-related hyperglycemia will increase AGE levels and active RAGE signaling to alter migration contributing to the progression of LV remodeling. By generating therapeutics against modulators of the RAGE signaling pathway, such as Rap1a, there is the potential to lessen diabetic complications.

## Data Availability Statement

All relevant data are included within the manuscript. Supplemental and original blot images are available: 10.6084/m9.figshare.10248983.

## Ethics Statement

The animal study was reviewed and approved by the University of Mississippi Institutional Animal Care and Use Committee.

## Author Contributions

All authors contributed to the experimental design, data analysis, and manuscript preparation. SB and MH conducted the experiments. JS obtained the funding.

## Conflict of Interest

The authors declare that the research was conducted in the absence of any commercial or financial relationships that could be construed as a potential conflict of interest.
